# Enhancing Anticorrosion Resistance of Aluminum Alloys Using Femtosecond Laser-Based Surface Structuring and Coating

**DOI:** 10.3390/nano13040644

**Published:** 2023-02-06

**Authors:** Tahir Nawaz, Asghar Ali, Shahbaz Ahmad, Piotr Piatkowski, Ali S. Alnaser

**Affiliations:** 1 Department of Physics, American University of Sharjah, Sharjah 26666, United Arab Emirates; 2 Materials Science and Engineering Program, College of Arts and Sciences, American University of Sharjah, Sharjah 26666, United Arab Emirates

**Keywords:** femtosecond laser nanostructuring, corrosion, alloys, coating

## Abstract

We report a robust two-step method for developing adherent and anticorrosive molybdenum (Mo)-based coatings over an aluminum (Al) 6061 alloy substrate using a femtosecond (fs) laser. The fs laser nanostructuring of Al 6061 alloy in air gives rise to regular arrays of microgrooves exhibiting superhydrophilic surface properties. The microstructured surface is further coated with an Mo layer using the fs-pulsed laser deposition (fs-PLD) technique. The combination of the two femtosecond laser surface treatments (microstructuring followed by coating) enabled the development of a highly corrosion-resistant surface, with a corrosion current of magnitude less than that of the pristine, the only structured, and the annealed alloy samples. The underlying mechanism is attributed to the laser-assisted formation of highly rough hierarchical oxide structures on the Al 6061 surface along with post heat treatment, which passivates the surface and provide the necessary platform for firm adhesion for Mo coating. Our results reveal that the corrosive nature of the Al-based alloys can be controlled and improved using a combined approach of femtosecond laser-based surface structuring and coating.

## 1. Introduction

Corrosion is a natural process responsible for transforming metals back to the ore from which they are extracted [1]. It is a result of the chemical or electrochemical interaction of metals with their environments that creates an impervious oxidation state on the surface and causes severe material degradation. A component or system’s functionality such as tensile strength, ductility, etc., is degraded as a result of the corrosion reaction, which transforms the original metal into a less desirable form [1]. Furthermore, the corrosion in metals can cause instrument failure, reduction in equipment lifetime, and safety hazards, especially in harsh conditions such as marine environments [2,3]. Globally, corrosion costs around USD 2.5 trillion per annum. By employing suitable corrosion suppression strategies, savings up to 35% of the corrosion losses could be realized. Protecting metals from corrosion is one of the most pressing challenges of the twenty-first century [1]. To address the present need for lightweight and durable components for technological applications, the development of high-strength, lightweight, and corrosion-resistant materials is necessary [4]. 

Alloys are materials formed from the combination of two or more metals to obtain a metallic material with desired properties, such as superior anticorrosion properties, a higher weight to strength ratio, etc. [5]. Alloys are favored against corrosion because of their heterogenous nature that reduces the corrosion and can result in high strength materials [6]. Among the different alloy systems, aluminum (Al)-based alloys are extensively utilized in automobile, aviation, and maritime applications due to their outstanding strength-to-weight ratios [7,8,9,10,11]. In Al alloys, a strong passive layer of Al_2_O_3_ forms on the surface as a result of the surface oxidation in an oxygen-rich environment. This Al_2_O_3_ passive layer is strongly bonded to the surface and if destroyed re-forms promptly in most cases. The protective Al_2_O_3_ layer is extremely stable and results in good pitting resistance in near-neutral solutions (pH 4–8.5) of most non-halide salts [12,13,14]. However, media that contain aggressive ions such as acidic media, alkaline solutions, and aqueous halide salt solutions deteriorate the corrosion inhibition properties of Al alloys [15,16,17,18,19,20]. The consequent pitting and intergranular corrosion on the surface may initiate cracks and thus lead to catastrophic component failure. This puts a limit on the potential applications of Al alloys in such aggressive environments [21,22,23].

For improving the corrosion resistance of surfaces, a number of physical and chemical techniques including anodizing [24], thermal spraying [25], chemical deposition [26], galvanizing [27], and hybrid coatings [28], etc., has been devised. Though these technologies are important, environmental unfriendliness, high-costs, and resource consumption cannot be overlooked [3]. Moreover, rapid development, coating uniformity, and good adhesion are the main technological imperatives.

Recently, ultrafast lasers, i.e., femto- and picosecond (fs and ps) lasers have garnered immense interest in surface engineering [29,30,31]. Because of their intense short pulses capable of delivering large amounts of power without causing significant damage to the region, neighboring the modified spot area, these lasers are frequently employed for controlled surface structuring. In addition to surface structuring, these lasers are proven tools for producing robust coatings for a host of technological applications [30]. The key laser parameters for producing such functional surfaces are the central laser wavelength, pulse duration, pulse fluence, repetition rate, scan type, scan rate, and line spacing [30,32].

Different studies have been reported on assessing the role of ultrafast lasers in producing functional surfaces for wettability transitions and corrosion inhibition [33,34,35,36,37,38]. Ahuir-Torres et al. [24] reported no change in the corrosion inhibition of AA2024-T3 after texturing with a ps laser. However, a change in wettability influenced by different surface structures was evidenced in the study. Some structures exhibited hydrophilicity, whereas other structures depicted a shift from hydrophilic to hydrophobic. Similarly, James et al. [25] demonstrated that fs laser-treated Al alloy degraded in corrosion inhibition relative to the untreated sample. The fs laser-processed samples suffered from pitting corrosion such that the corrosion rate was faster than that of the untreated sample. Similarly, they demonstrated that superhydrophobicity achieved with laser structuring alone is inefficient in suppressing corrosion. Yuan et al. [39] reported that femtosecond laser-treated samples require quite a long aging time (around 30 days) to transform superhydrophilic to superhydrophobic surfaces; however, they suggested that thermal treatments can rapidly transform the laser-treated surface to a superhydrophobic surface. Similarly, they reported self-healing and anticorrosion properties after the suggested thermal treatment. In contrast, Rajan et al. [17] found out that fs laser-treated Al 6061 depicted a corrosion rate two orders of magnitude smaller than that of the untreated sample. Here again, thermal treatment was reported to transform the surface into a superhydrophobic surface. However, as reported in [22,40,41], the superhydrophobic surfaces convert into hydrophilic surfaces after some time, especially when immersed in an aqueous medium. Thus, the corrosion suppression solely due to superhydrophobicity cannot be relied upon for long-term immersion in aqueous solutions (e.g., sea water). Therefore, an extra layer of a corrosion suppressant that can enhance the corrosion inhibition of Al 6061 would be needed.

Due to their excellent chemical, mechanical, and electrochemical attributes, transition metals and their alloys have been in focus for many years [42,43,44,45]. In addition to corrosion inhibition, transition metals, their alloys, and transition-metal based composite coatings have been employed for enhancing other surface properties such as wear resistance [46,47,48,49,50], hardness [51,52,53,54], erosion [55,56,57,58], tribology [59,60,61,62,63], etc. Jianhui Yan et al. [46] analyzed the changes in the microhardness and the wear resistance of Mo and Mo-MoSi_2_-based composite coatings prepared through the atmospheric plasma spraying process. Similarly, J.F. Santa et al. [55] reported that the erosion of steel could be improved up to 16 times through Ni, Cr, and WC coatings applied on the steel surface. Likewise, Ali et al. demonstrated that an fs-PLD-based Mo oxide coating improved the corrosion inhibition in aqueous NaCL by 95% relative to polished mild steel [30].

In this work, we analyze the effects of surface structuring and thermal treatments on the wettability transition and corrosion inhibition of Al 6061. The effects of an Mo oxide coating on improving the corrosion inhibition of Al6061 are also assessed. We employ an fs laser to produce hierarchical micro/nanostructures on Al 6061, and we analyze the impact of surface structuring on wettability transition and corrosion inhibition. The role of thermal treatment in inducing wettability transitions and corrosion inhibition is also evaluated. This is followed by investigating the effects of an Mo oxide coating on the corrosion resistance of laser-structured Al 6061. For this purpose, a thick Mo oxide coating is developed on structured Al 6061 with fs-PLD and subjected to corrosion studies in 3.5 wt% NaCl solution.

## 2. Methodology

In this study, three sets of Al 6061 alloy samples were prepared. All three sets were surface nanostructured with a femtosecond laser. Of the three sets, one was used as only laser structured (L), one set was laser structured and heat-treated (annealed) (L + H), and the other one was coated after laser structuring (L + C). The current study used an Active Fiber Systems Ytterbium-300 laser (model AFS-UFFL-300-2000-1030-300 from Active Fiber Systems GmbH, Jena, Germany) with an overall combined power output of 300 W and output wavelength of 1030 nm.

For nano-structuring of the sample surfaces, a 50 kHz femtosecond laser beam with a pulse duration of 40 fs, laser fluence of 6 J/cm^2^ to 15 J/cm^2^, 50µm spot size, and 100 µm step size was used. A half-wave plate (HWP) and a thin-film polarizer (TFP) were used in tandem to adjust the laser power and provide a single pulse with 120 μJ of energy. The attenuated laser beam was then focused on the Al6061 substrate using the scan head, an F-Theta lens, and a raster scanner (FARO tech. Xtreme-20, Faro Technologies, Inc., Lake Mary, FL, USA). The scanning speed was set to 20 mm/s, and the spot overlap rate was 99.2%. The crosshatch structuring was performed with 3 cycles each on all structured samples. After ablation, the samples were sonicated in DI water for 1 h and dried with compressed air afterwards. One set of the samples was annealed at 400 °C for 10 h in an electric furnace.

**Figure 1:**
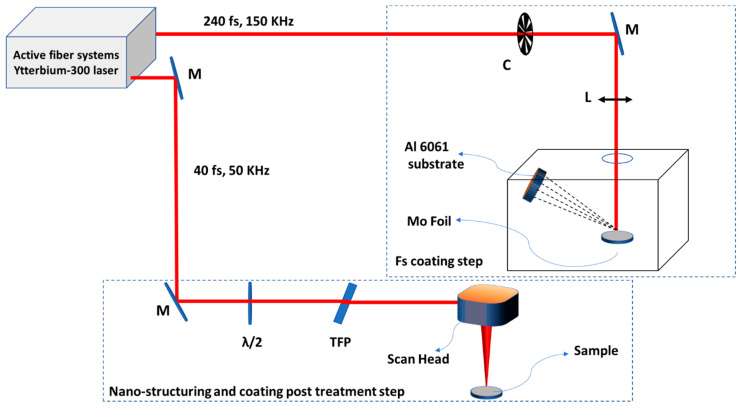
Schematic diagram of the experimental setup: Step 1—femtosecond pulsed laser deposition of the Mo over the substrate (with 240 fs, 150 kHz laser beam), Step 2—post treatment of the sample in step 1 using a scan head (with 40 fs and 50 kHz laser beam), C—optical chopper, M—dielectric mirror, L—objective lens, λ/2—half wave plate, TFP—thin film polarizer.

The coating process was performed in 2 separate steps: (1) in the first step, a laser beam with a pulse duration of 240 fs, fluence of 3 J/cm^2^, and frequency of 50 kHz was used to deposit Mo on the sample surface in a vacuum. The beam was focused onto a rotating cylinder with several layers of Mo foil wrapped around it. (2) After deposition, the sample was post treated with a laser beam having a pulse duration of 40 fs, frequency of 50 kHz, wavelength of 1030 nm, fluence of approximately 1.5 J/cm^2^ and 0.25 J/cm^2^, scan rate of 3325 mm/s, and step size of 10 µm using a scan head. The process was performed 4–6 times with a 10 min deposition time interval. Figure 1 shows the schematic diagram of the experimental setup for nanostructuring and deposition processes. All the parameters for laser processing are summarized in Table 1.

The electrochemical tests were performed using a CH Instruments 660E workstation in 3.5% NaCl solution. A tri-electrode setup with Pt as the wire counter electrode and Ag/AgCl reference electrodes was used. The exposed surface area of the working electrode was 1 cm^2^. The open circuit potential (OCP) tests were performed for 2 h on all samples to obtain a stable electrochemical interface. A scan rate of 10 mV and auto sensitivity settings were employed to obtain the Tafel plots (cyclic polarization curves). A negative voltage window of −1.8 to 0 was employed for the cyclic polarization tests. Electrochemical impedance spectroscopy (EIS) analyses were performed at OCP in a frequency range of 100 Hz–1 M Hz and 10 mV excitation amplitude.

The morphological and elemental studies were carried out using a TESCAN VEGA 3 LMU scanning electron microscope and an INCAx-act EDS detector from Oxford Instruments. SEM analysis was performed for structural morphology. For a detailed crystallographic and phase study, X-ray diffraction analysis was conducted with Bruker’s D8 Advance diffractometer with 2θ ranging from 10° to 90° with a high resolution of 0.05°/min steps and time per step of 0.5 s. A Renishaw inVia confocal Raman microscope was used to obtain information regarding the chemical nature of the samples’ surfaces. An excitation wavelength of 514 nm and power of 1.5 xmW were used. Surface wettability tests for water contact angle (WCA) measurement were conducted using a Drop Shape Analyzer (DSA-100, KRUSS, Matthews, NC, USA).

## 3. Results and Discussion

**Figure 2:**
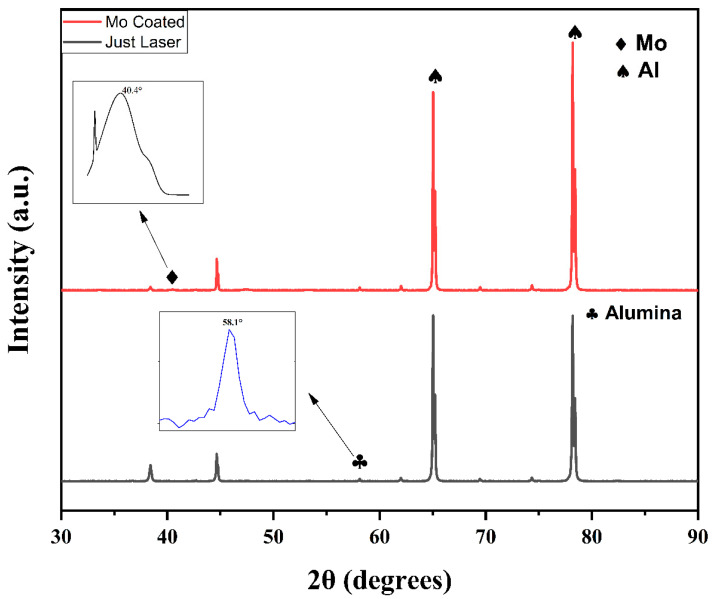
XRD peaks for just laser-structured samples showing major peaks of Al and a small alumina peak at 58.1°; and laser-structured and Mo-coated sample showing the small peak of Mo.

For the detailed crystallographic and phase study, X-ray diffraction analysis was conducted with Bruker’s D8 Advance diffractometer with 2θ ranging from 10° to 90° with a high resolution of 0.05°/min steps and time per step of 0.5 s. The peaks were observed at 38°, 46°, 65°, and 79° for all samples, as shown in Figure 2. From the literature, these peaks belongs to Al. The peaks for the thermally untreated, thermally treated at 400 °C, and the Mo-coated samples were observed at the same angle positions. The only difference observed was the very slight shift in the intensity of the peaks for the 400 °C-treated samples [64]. This could be attributed to the high temperature treatment, as 400 °C is the crystallization temperature for Al and most of its alloys. However, a very minor peak of alumina was observed at around 58°. This might be attributed to the fact that the fast scanning speed for laser nanostructuring does not allow for the proper development of a crystalline structure. Therefore, an amorphous or a short-range nano-crystalline structure was observed [65]. Rajan et al. [22] reported that fs laser structuring forms alumina with very small intensity peaks, thus depicting partially crystallinity. Similarly, no long-range order was observed for the Mo-based sample. A very small Mo peak at 40.2° was observed, which is indicative of preferential (110) cubic Mo alignment [66]. The presence of Mo and its oxides was also confirmed by Raman analysis, which is discussed below.

**Figure 3:**
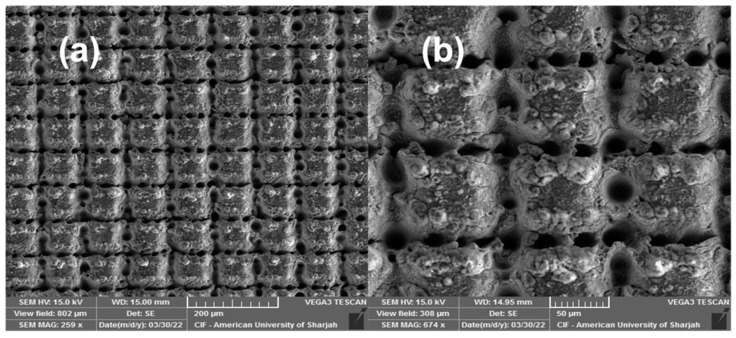
SEM images of laser-structured surfaces: (**a**,**b**) formation of microgroove structures on the Al 6061 surface as a result of femtosecond laser ablation.

Figure 3 shows the surface morphology of the 6W laser-structured Al 6061 sample. Hierarchical structures comprising cross-hatched microgroove structures superimposed by submicron- and nanostructures are shown in Figure 3a,b. Similar structures were reported previously in [22,67]. Those structures result from multiple laser scans that are responsible for deepening the grooves with each subsequent scan. When a high-power focused laser pulse interacts with a surface, local heating followed by instantaneous cooling takes place. The pulse fluence determines the ablation rate and the penetration depth [68], where considerable ablation takes place for fluence beyond the ablation threshold [30]. Similarly, the accumulated fluence determines the morphology and other properties of the surface features that emerge [65]. Excessive melt hydrodynamic movement and explosive material removal result from ablation at high fluence. The ablation debris that flies off during the ablation process as well as the vaporized species in the plasma that weld together upon collision give rise to these submicron and nanofeatures observed in Figure 3 [30]. The reason that laser-induced periodic surface structures (LIPSS) did not predominantly occupy the surface is that LIPSS tend to appear on stationary cooling melt. Since 6 W of power induced excessive ablation along with strong hydrodynamic movement, LIPSS structures did not appear covering the surface. Furthermore, surface treatment in oxygen-rich environments has been reported previously to cause surface oxidation, grain refinement, and increased stress [69].

To develop Mo-based coatings on top of the crosshatched microgroove-structured Al 6061, we employed an all fs-PLD process. We recently developed this technique for producing high quality refractory metal oxide coatings [30]. The technique involves a raw deposition step followed by subsequent high-speed scanning in ambient atmosphere to produce refractory metal oxides. In this study, we employed this technique to develop an Mo oxide coating on top of the laser-structured Al 6061.

**Figure 4:**
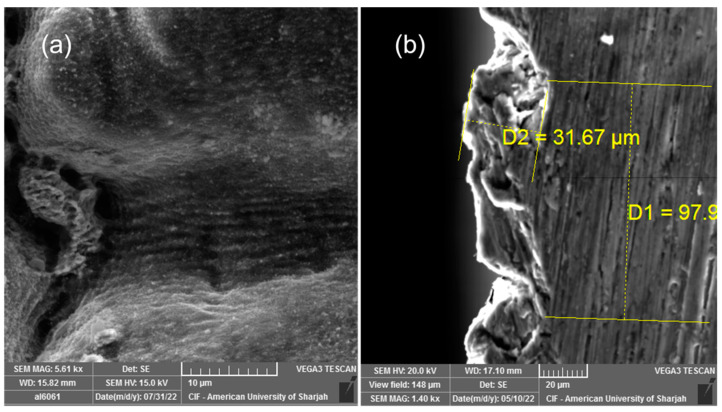
SEM micrograph of the Mo coated on Al 6061 substrate: (**a**) Mo coating with post laser surface treatment with 1.5 W and 0.25 W; (**b**) cross section of the deposited Mo coating over Al 6061 substrate after laser treatment with an average final thickness of D2 of ~30 microns. D1 represents the step size of the laser structuring.

Figure 4a depicts the Mo oxide-coated cross-hatched groove structure. Micron and submicron features are observed in the micrograph. Though some ablation debris can still be observed along the surface, other types of structures also emerged after the Mo deposition. Figure 4b shows the cross section of the Mo oxide-coated sample. By virtue of the fs-PLD process, a thick Mo oxide layer was deposited. An average Mo-based thickness of around 30 μm covering the sample was formed.

**Figure 5:**
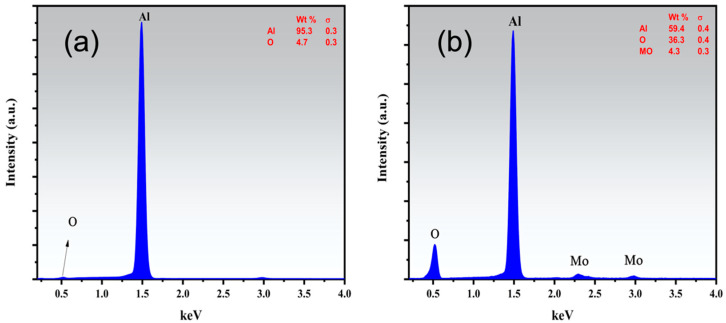
EDS spectra of (**a**) pristine Al 6061 and (**b**) MoO_(x)_-coated structured Al 6061. The O content is around 5 wt% in the pristine sample. The coated sample shows Mo presence as well as an enhanced O content of over 30 wt%.

Figure 5 depicts the elemental analysis of the untreated pristine Al 6061 and the Mo oxide-coated Al 6061. The EDS confirmed the presence of Mo along the underlying Al 6061. Moreover, an O content of more than 30% was also observed in the Mo-coated structured sample. A thick layer of oxides was formed when multiple-fold laser treatment was performed on the material surface [70,71]. This was attributed to the fact that a multiple-fold treatment maintained the reactive state of the surface for a longer period of time, thus facilitating more oxidation. Moreover, the repeated melting and solidifying of the surface resulted in a thick layer of oxides covering the surface [71,72]. Furthermore, the O content along the surface and into the coating was also expected to increase because of facilitated melt convection and O diffusion [30].

**Figure 6:**
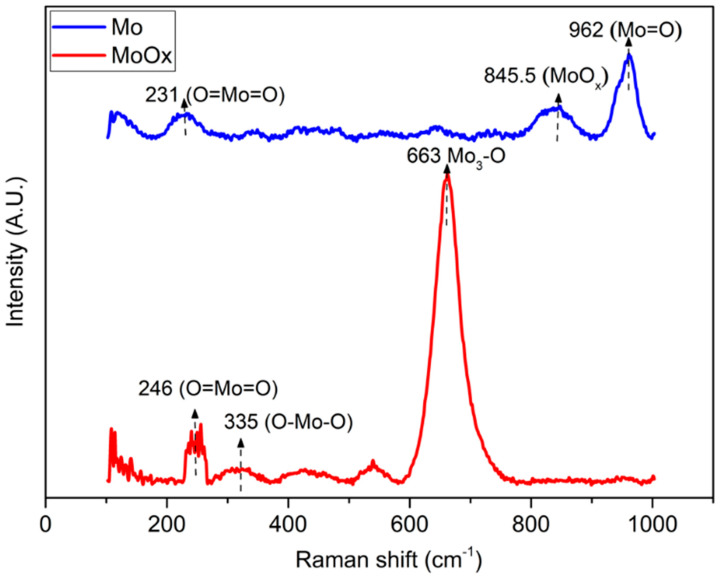
Raman shift of the as-deposited Mo, i.e., without post laser treatment showing the Mo deposited on the surface (this process is in vacuum and metallic Mo is expected); post deposition laser treated with 1.5 and 0.2 W showing the formation of oxides MoO_x_ on the surface of the Mo-coated sample (this process is in air, and oxidation is expected).

Figure 6 shows the Raman spectra of pristine Mo and its oxide coatings (MoOx) deposited at the laser-structured (L + C) sample. Several peaks for Mo and MoOx, but in particular the high-intensity peak at 663 cm^−1^ for MoOx, were attributed to the phonon stretching vibration of crystalline α-MoO_3_ with an orthorhombic layer structure [73,74,75,76]. The Raman peaks at 335 and 845.5 cm^−1^ were due to the O–Mo–O bending mode, which is an intermediate oxidation state. This intermediate oxidation state is MoOx where 2 < x < 3, which means that the compound can be oxidized further to the MoO_3_ state by changing the laser parameters [77]. The peaks at 231 and 246 cm^−1^ could be assigned to the twisting and wagging modes of O=Mo=O bonds [78].

**Figure 7:**
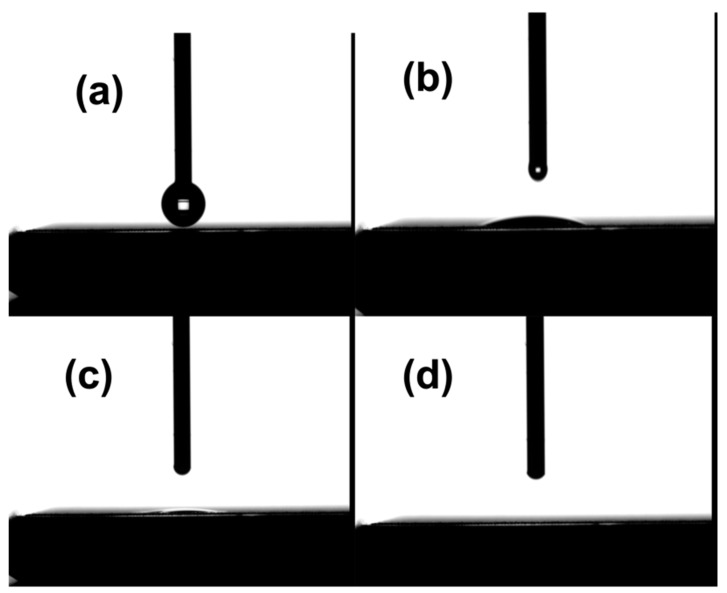
(**a**–**d**) Water contact angle test of the laser-treated surfaces showing superhydrophilic properties. From (**a**–**d**), the images show the frame by frame formation of a drop contacting the surface and dispersing on the laser-treated surface within a fraction of a second.

Figure 7 shows the wettability test of the treated (all three sets) samples. All the samples were tested after laser structuring. The laser-structured and annealed samples were tested prior to and after the annealing process. The surfaces of just L and L + H samples showed superhydrophilic properties with a water drop spreading within a fraction of a second. This result was consistent with laser-structured samples regardless of their power. Similar results were shown by Zheng et al. [79]. They reported that the aluminum surface obtained through laser structuring showed superhydrophilicity and was attributed to the high surface roughness of the laser-treated surface. Our obtained results contradict the previously reported studies, in which the anti-corrosion performance of the crosshatch microgroove-like structures, obtained using femtosecond laser-surface structuring, was linked to superhydrophobicity [22,80,81]. The superhydrophobic behavior was attributed to the trapping of air molecules into the microstructures (Cassie–Baxter model), in addition to thermal aging and chemosorption of organic molecules on the surface [22,80,81].

**Figure 8:**
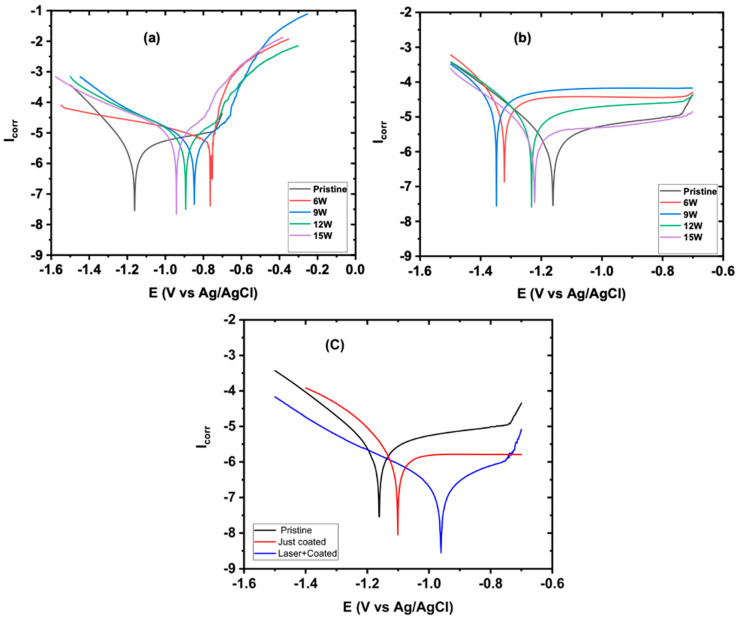
Tafel plots of (**a**) laser-treated and annealed samples, (**b**) just laser-treated samples, and (**c**) a comparison of the pristine, the only Mo-coated, and the laser-structured and Mo-coated samples.

The Tafel extrapolation method was used to determine the corrosion potential (*E_corr_*) and corrosion current (*I_corr_*) from the cathodic polarization curves. Figure 8a shows the polarization curves for the laser-structured and annealed samples. The results show that the annealing process produced a stable oxide layer, which caused passivation in the laser-structured samples with a positive shift in *E_corr_* for 6 W (0.5 V), 9 W (0.49 V), 12 W (0.34 V), and 15 W (0.28 V), respectively. However, there were very small changes in the *I_corr_*. This was due to the passive oxide layer that acted as a physical barrier to corrosion or further oxidation, thus decreasing the current density [82,83,84]. Furthermore, Figure 8b shows the laser-structured samples treated at laser powers of 6, 9, 12, and 15 Watts. Those samples demonstrated poor anticorrosive performance, associated with current densities of 22.2, 31.6, 7.53, and 3.79 μA/cm^2^, compared to the pristine sample (1.93 μA/cm^2^). These high *I_corr_* values of the laser-structured samples could be attributed to the increase in the effective surface area, which increased the electrode to electrolyte interface, thus increasing the sites of attack for the reactive ions present in the solution and leading to decreases in the corrosion resistance of the surface [84,85,86].

Though the annealing process induced passivation in the laser-treated samples by shifting the *E_corr_* during Tafel polarization in the positive direction, the minimal change in *I_corr_* suggested a genuine need for a protective coating to improve the corrosion inhibition efficiency. Figure 8c shows the Tafel plots for the coated sample without structuring and the laser-structured and annealed Al 6061 samples coated with Mo by the fs-PLD process. As shown in Figure 8c, the just-coated sample exhibited an *I_corr_* value of 0.89 μA/cm^2^ (compared to pristine sample of 1.93 μA/cm^2^) and showed a corrosion inhibition efficiency (*CIE*) of 53%. To further improve the *CIE*, the sample was laser treated prior to coating. This step induced a layer of oxides on the sample surface and then annealing. As a result, a thick passive layer of oxides of aluminum (as supported by EDS and XRD) was formed on the surface. These samples were then coated with Mo using the fs-PLD process. The laser and coated (L + C) sample exhibited an *I_corr_* value of 0.316 μA/cm^2^ with approximately an 83% increase in the corrosion inhibition efficiency (*CIE*), as calculated from Equation (1):

(1) $$CIE\%=\frac{I_{corr}-{I^{\prime }}_{corr}}{I_{corr}}\;\times \;100$$

where *I_corr_* and *I’_corr_* are the corrosion current densities before and after, respectively, the surface modifications and coating.

**Figure 9:**
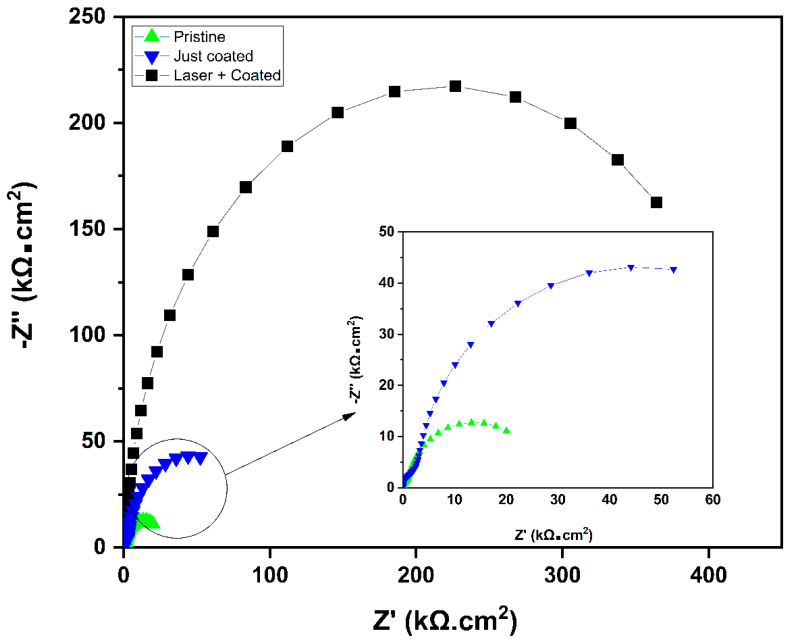
EIS analysis of pristine, just laser-structured, just-coated, and laser-structured with Mo-coated samples. The Nyquist plot of the laser-treated and coated samples have the largest diameter (i.e., greatest resistance) compared to the just-coated as well as pristine samples.

From Ohm’s law, *I_corr_* = E/R_p_, where E is the potential difference between the cathode and anode on the potentiodynamic curve; R_p_ is the polarization resistance, which is the sum of the electric resistance of the corrosive solution (R_s_) and the charge transfer resistance of the working electrode (R_ct_). For the laser-treated and coated sample, the R_ct_ was modulated by the thick layer of the Mo coating along with the oxide layer of the substrate material surface induced using the femtosecond laser, while for the just-coated sample, the R_ct_ was modulated by the Mo-coated layer only. Both R_s_ and R_ct_ were determined using the Nyquist plots. As shown in Figure 9, the L + C sample showed the highest R_ct_ value of 431 kΩ compared to 82.8 kΩ and 23 kΩ of the just-coated and pristine samples, respectively. The high R_ct_ value of the L + C sample compared to the just Mo-coated sample proves the double layered protection with Al_2_O_3_ and Mo.

## 4. Conclusions

Al6061 alloy was investigated for its corrosion performance. Three sets of samples, i.e., only laser structured, laser structured and annealed, and laser structured and Mo-coated samples, were prepared and compared. Laser fluence ranging from 6 J/cm^2^ to 15 J/cm^2^ was used in the ablation process. Mo was deposited on the nanostructured Al alloy substrate using the femtosecond laser pulsed laser deposition process with better adhesion compared to unstructured substrate. The femtosecond laser ablation process induced a thick oxide layer over the substrate surface, while the Mo coating further enhanced its ability to withstand corrosive environments. The laser nano-structuring produced superhydrophilic surfaces, which have poor corrosion resistive performance despite having a thick oxide layer. It was observed that laser structuring alone leads to poor corrosion performance in NaCl environments due to the increase in the overall surface area of the sample, which requires another coating layer to enhance the corrosion inhibition. The Mo-coated samples showed improved corrosion inhibition performance compared to the pristine samples and the only structured samples.

## Figures and Tables

**Table 1:** Laser parameters for nano-structuring and coating.

Laser Parameter	Value
Nano-Structuring	
Fluence range	5–15 J/cm^2^
Frequency	50 kHz
Scanning speed	20 mm/s
Pulse duration	40 fs
Step size	100 µm
Beam wavelength	1030 nm
Coating and post treatment	
Fluence	3 J/cm^2^ for deposition process, 1.5, and 0.25 J/cm^2^ for post-deposition treatment
Frequency	150 kHz for deposition and 50 kHz for post-deposition treatment
Scanning speed	3325 mm/s for post-deposition treatment
Pulse duration	240 fs for deposition process and 40 fs for post-deposition treatment
Step size	10 µm for post-deposition treatment
Beam wavelength	1030 nm

## Data Availability

Any further details relevant to this study may be obtained from the authors upon a reasonable request.
